# The Role of MicroRNA 143 and MicroRNA 206 in The Regulation of Apoptosis in Mouse Lukemia Cancer Cells and Spermatogonial Cells

**DOI:** 10.22074/cellj.2021.7606

**Published:** 2021-10-30

**Authors:** Azar Shams, Ronak Shabani, Mohammad Najafi, Mahdi Karimi, Vahid Pirhajati, Mohammad Asghari Jafarabadi, Hamid Reza Asgari, Chad B. Maki, Seyed Mohsen Razavi, Morteza Koruji

**Affiliations:** 1. Stem Cell and Regenerative Medicine Research Center, Iran University of Medical Sciences, Tehran, Iran; 2. Department of Anatomical Sciences, School of Medicine, Iran University of Medical Sciences, Tehran, Iran; 3. Department of Biochemistry, School of Medicine, Iran University of Medical Sciences, Tehran, Iran; 4. Department of Nanotechnology, School of Medicine, Iran University of Medical Sciences, Tehran, Iran; 5. Neuroscience Research Center, Iran University of Medical Sciences, Tehran, Iran; 6. Department of Statistics and Epidemiology, School of Medicine, Zanjan University of Medical Sciences, Zanjan, Iran; 7. Center for The Development of Interdisciplinary Research in Islamic Sciences and Health Sciences, Tabriz University of Medical Sciences, Tabriz, Iran; 8. VetCell Therapeutics USA, 2917 Daimler Street, Santa Ana CA 92705, USA; 9. Oncopathology Research Center, Faculty of Medicine, Iran University of Medical Sciences, Tehran, Iran

**Keywords:** Apoptosis, Cancer, MicroRNA, Poly Lactic-Co-Glycolic Acid, Smart Gene Delivery

## Abstract

**Objective:**

In cancer treatments, smart gene delivery via nanoparticles (NPs) can be targeted for cancer cells, while concurrently minimizing damage to healthy cells. This study assessed the efficiency of poly lactic-co-glycolic acid (PLGA)-miR 143/206 transfection on apoptosis in mouse leukemia cancer cells (El4) and spermatogonial stem cells (SSCs).

**Materials and Methods:**

In this experimental study, neonatal mouse spermatogonia cells and EL4 cancer cell lines were used. MicroRNA-PLGA NPs were prepared, characterized, and targeted with folate. Several doses were evaluated to obtain a suitable miR dose that can induce appropriate apoptosis in EL4 cells, while not harming SSCs. Cells were treated separately at 3 doses of each miR (for miR 143, doses of 25, 50 and 75 nmol and for miR 206, doses of 50, 100 and 150 nmol). The experiments were performed at 24, 48 and 72 hours. Viability and apoptosis were investigated by MTT and Annexin Kits.

**Results:**

Based on MTT assay results, the optimal dose of miR 143 was 75 nmol (59.87 ± 2.85 % SSC and 35.3 ± 0.78 % EL4) (P≤0.05), and for miR 206, the optimal dose was 150 nmol (54.82 ± 6.7 % SSC and 33.92 ± 3.01% EL4) (P≤0.05). The optimal time was 48 hours. At these doses, the survival rate of the EL4 cells was below the half maximal inhibitory concentration (IC_50_) and SSC survival was above 50%. Annexin V staining also confirmed the selected doses (for miR 143 total apoptosis was 6.62% ± 1.8 SSC and 37.4% ± 4.2 EL4 (P≤0.05), and miR 206 was (10.98% ± 1.5 SSC and 36.4% ± 3.7 EL4, P≤0.05).

**Conclusion:**

Using intelligent transfection by NPs, we were able to induce apoptosis on EL4 cells and maintain acceptable SSC survival rates.

## Introduction

Chemotherapy and radiotherapy cause serious damage to spermatogonial stem cells (SSCs), resulting in early loss of SSCs and temporary infertility. The proportion of azoospermic as late effect of cancer was reported 18%, specifically for leukaemias (1).

Today, there are promising ways to manage the consequences of infertility in adulthood such as transplantation of SSCs (2) or testicular tissue transplantation (3). Adult males will have a chance to maintain fertility through ejaculation and cryogenic preservation before starting treatment, but this is not an option in pre-puberty. A promising method to preserve fertility in children with cancer is via testicular biopsy before the onset of cancer treatment, followed by isolation, proliferation, maintenance and transplantation (4, 5). But, a major concern with this method is the possibility of tissue contamination with cancer cells which introduces the risk of recurrence of the cancer (6, 7). At this time, magnetic-activated cell sorting (MACS) and fluorescence-activated cell sorting (FACS) are the most utilized techniques to eliminate cancerous cells, but they are not sensitive enough to completely eliminate tumor cells (8). Because of different spermatogonial cell markers as well as the variability of cell size, the efficiency of MACS has lessened and the survivability of cells with this method is questionable (9). Therefore, rather than focusing on positive SSC selection, the development of new methods for eliminating cancer cells can provide a more effective solution.

Nanoparticles (NPs), which have the ability to deliver targeted therapies to specific cell types through their structural changes and physical reformation, (change in their shape, size, and physical and chemical properties), have increased the success rate of a new therapeutic strategy (10). Many microRNAs (MiRs) are used to induce apoptosis in cancer cells. MiRs are subgroups of noncoding RNAs that are about 20-25 nucleotides long and affect the expression of genes after transcription, among these MiR 143 and MiR 206 play a more prominent role. Many studies have shown the effects of these MiRs on induction of apoptosis in cervical cancer cells (11), oral squamous cells (12) and human epithelial cells (13). Transfection of MiRs via NPs in order to induce apoptosis has been very promising. Among the NPs, poly lactic-co-glycolic acid (PLGA), due to its high biocompatibility and bioavailability, non-toxicity, non-immunogenicity and food and drug administration (FDA) approval, is used extensively (14). In addition, targeting drug carriers to cancer cells can increase treatment efficacy. Conjugated NPs with folic acid can facilitate the entry of NPs into cells (15). Cancer cells have large amounts of folic acid receptors on the surface of their cell membranes, which makes it easier to target NPs to this type of cell as compared with normal or non-cancerous cells (16).

In human testicular biopsy samples, there is no threshold for the risk of recurrence in the form of a transplant, and this ambiguity requires more sensitivity to clean up and purify the SSCs. So far, no studies have measured the effects of these apoptotic inducers on cancer and spermatogonia cells from a fertility perspective. Therefore, we assessed the efficiency of PLGA- MiR transfection on apoptosis in mouse leukemia cancer cells (El4 cells) and SSCs to determine the optimal effective dose to eliminate EL4 cells, while maintaining adequate SSC viability.

## Materials and Methods

In this experimental study, MiR 143 and MiR 206, including primer and fluorescent marker (FAM), were purchased from pishgam co. Sequence data for these have been submitted to the GenBank databases under accession numbers (Mir 206: MI0000490, Mir 143: NR_029684). PLGA (Resomer RG502H), with a 50:50 mole ratio of glycolic acid to lactic acid and a molecular weight of 12,000 g/mol and polyvinyl alcohol (89 mol% hydrolyzed) (Sigma, St Louis, MO, USA) same as previous study (17). 

Fifty neonatal NMRI mice 3-6 days were used to extract spermatogonia cells. The research was approved by the Research Ethics Committee of Iran University of Medical Sciences (IR.IUMS.FMD.REC.1396.9321113001). 

### Cancerous cell line (EL4 cells)

EL4 cancer cell line was purchased from the Pasteur Institute (Tehran, Iran). To confirm cell line characteristics, flow cytometry with a H-2kb specific marker was used (Abcam, UK). Based on our perivous studies (17, 18), EL4 cells were cultured at 37˚C in a 5% CO_2_ , in DMEM/F12 (Gibco, USA) with 2% FBS, penicillin (100 U/mL) and streptomycin (100 µg/mL). To evaluate the functionalities of EL4 cells and their tumorigenicity, 5×10^5^ cells in a volume of 10 μl was injected with a suitable diameter needle (70 μm) and under a stereomicroscope (Olympus, SZ1145, Japan) through the efferent ductile, rete testis and ultimately into the seminiferous tubules in mouse reciepent.

### Spermatogonial stem cell culture

A total of 50 NMRI neonate mice were used for SSC extraction. Testicles were kept on ice after separation from the animal until they were transferred to the culture medium. After washing in PBS and DMEM/F12, the testes capsules were isolated. After two phases of enzymatic digestion with the enzymes hyaluronidase (1 mg/ml), collagenase IV (4 mg/ml) and trypsin (0.25%), SSCs were extracted and cultured for 2 weeks. 

To confirm SSC phenotype, flow cytometry analysis with Plzf marker with Alexa flour anti-mouse Plzf antibody was used (biosciences). Additionally, polymerase chain reaction (PCR) was used to examine specific genes associated with SSCs (*Plzf, Gfrα1, Oct4* and *Gapdh* as a housekeeping gene).

*Oct4-*

F: 5ˊGAACTAGCATTGAGAACCGT3ˊ

R: 5ˊCATACTCGAACCACATCCTTC3ˊ

*Plzf-*

F: 5ˊCCCGTTGGGGGTCAGCTAGAA3ˊ

R: 5ˊCTGCAAGGTGGGGCGGTGTAG3ˊ

*Gfrα1-*

F: 5ˊAATTGTCTGCGTATCTACTGG3ˊ

R: 5ˊACATCTGATATGAACGGGAC3ˊ

*Gapdh-*

F: 5ˊCTGCTGGACAAGTGAGTCCC3ˊ

R: 5ˊCCAAGTACCCTGGCCTCATC3ˊ

Total RNA were extracted using RNA extraction kit (Qiagen, Germany) besed on the manufacturer’s instructions. RNA was checked by a 260/280 nm ratio measurement, Reverse-transcription PCR (RT-PCR) was done by using complementary deoxyribonucleic acid (cDNA), primers and with PCR Master Mix 2X kit (Fermentas, St. Leon-Rot, Germany). To calculate gene expression Gene Runner software (version 3.02; Hastings Software Inc, New York, NY, USA) was used. The following conditions were used: Incubation at 95˚Cfor 3 minutes, denaturation for 30 seconds at 95˚C, Annealing for 30 seconds at the specific temperature associated with each primer and extension for 1 minute at 72˚C. After completion of the reaction, 5-10 μl of PCR solution on 1.2% agarose gel was analysed.

### Preparing MiR-PLGA

In order to encapsulate MiR in the PLGA polymer structure, emulsion-solvent penetration was used. Tween 80 and span 80 surfactants were used as emulsifying agents. Polyvinyl alcohol was used as a stabilizer. Briefly, it should be noted that all appliances were deprotected with DEPC-treated water and glass containers were baked for 4 hours at 240˚C in order to remove any RNase enzymes. In order to obtain the desired Nano capsule, the internal water phase was initiated by the formation of polyethyleneimine (PEI) 25KDa and MiR with a mass ratio of 3 to 1. In practice, however, different ratios of PEI were used at this stage and eventually a synthesis was selected that had the appropriate zeta potential and was capable of loading the minimal genetic material required by agarose gel retention test. At first, a 0.1% solution of PEI was prepared in DEPC water. The MiR solution was also prepared using DEPC water at a concentration of 100 picomoles per microliter. Then, 80 μl of PEI containing 90 μg of PEI and 40 μl of MiR containing 30 μg of MiR were combined and the volume was adjusted to 500 μl PBS and maintained for 30 minutes in a thermal shaker at 37˚C. Because MiR was labeled, the entire process was performed in the dark.

To prepare the organic phase in solvent evaporation, 10 mg of PLGA was dissolved in 2 ml of organic solvent of ethyl acetate. To form the initial emulsion, 500 μl of internal aqueous phase with 0.5 ml of span 80 solutions at a concentration of 5 mg/ml was added to the organic phase and, using a vortex and ultrasonic bath, an initial water emulsion in oil was created. In the final step, 4 ml of PVA 0.5% was added and was agitated with a magnetic stirrer for 4 hours. In order to separate and purify, centrifugation at 12,000 rpm for 30 minutes and washing steps were performed two times and were finally resuspended in distilled water. This was performed so that the polyplexes were not loaded and the extra surfactants in the aqueous phase were removed from the surface of the NPs.

### Surface modification of MiR-PLGA with folate

One mmol of folic acid in 20 ml DMSO with 1 mmol of ethyl dimethylaminopropyl carbodiimide (EDC) and 2 mmol of N-Hydroxysuccinimide (NHS) was dissolved in with a magnetic stirrer for 12 hours at room temperature. Then, to remove excess material, the solution was filtered and added to the ethylenediamine (EDA) as a linker, and pyridine (0.2/ml) and acetonitrile added to precipitate the folate. The precipitate was washed 3 times with diethyl ether and then dried to give a yellow deposit. 10 mg of MiR-PLGA was dissolved in 5 ml phosphate-buffered saline (PBS, Abcam, UK) at pH=7.4 and sonicated for 1 minute. 1 mg/ml EDC and 1 mg/ml of NHS were dissolved in DW and each of them washed, and about 500 μl was added to the above solution and agitated with a magenetic stirrer at room temperature for 2 hours and then centrifuged for 20 minutes to remove EDC and NHS. Following, folic acid was added to the product with the ratio of 1 mg/ml in PBS, incubated overnight at room temperature, agitated with the magenetic stirrer and then centrifuged. Using the same procedure as a previous study (17) the obtained solution was mixeded at room temperature once more and then the extra non-conjugated material was picked up by ultracentrifugation. Finally, the residual solution was lyophilized and stored. Conjugation of folate on the PLGA surface was confirmed by fourier transform infrared spectroscopy (FTIR) analysis.

### The dynamic light scattering

A portion of dried powder of MiR-PLGA was dissolved in 1 ml of PBS with pH=7.4, using ultrasonic bath. Zeta potential was measured using a zeta meter device. 

### Transmission electron microscopy

The shape of NPs were evaluated by transmission electron microscopy (TEM, LEO 906; Carl Zeiss). The solution was sonicated for 5 minutes, then one drop of NP suspension (1 mg/mL) was put on a carbon-coated copper TEM grid and dried in the air. Finally,the sample was imaged by a KV 100.

### Fourier transform infrared spectroscopy analysis

Using FTIR, the chemical structure of the NPs was studied. The test was carried out on 3 samples including polymer, folic acid and loaded NPs with a surface texture with folic acid. Approximately 2-3 milligrams of sample with potassium bromide powder was converted to a tablet using a 12-ppm hydraulic press and then FTIR spectra were collected. 

### Evaluation of miR-PLGA-folic acid uptake

Uptake was measured by fluorescence of MiR-PLGA incubated with EL4 cells in the culture medium.

### Dosimetry

Due to the fact that treatment with MiR -PLGA-Folic acid was performed on SSCs as healthy cells, it was necessary to obtain a optimal MiR dose that can induce appropriate apoptosis in EL4 cells, but remain non-harmful to SSCs. So several doses were evaluated in the dosimetry stage (based on several previous studies (19, 20). Cells (EL4s and SSCs) were treated separately at 3 doses (for MiR 143, 25, 50 and 75 nmol and for MiR 206, 50, 100 and 150 nmol). The experiments were performed at 24, 48 and 72 hrs to investigate the most optimal time for transfection. Each experiment was repeated 3 times.

### Cytotoxicity assay

MTT was used to assay the toxicity MiR-PLGA-Folic Acid and the survival rate of EL4 cells and SSCs. 2×10^4^ cells per well were seeded in two 96-well plates for each cell type. After treating with various doses (for MiR 143 : 25, 50, 75 nmol and for MiR 206: 50, 100, 150 nmol) the culture medium was extracted from the well and 10 μl/well MTT solution (5 mg/ml) was added, incubated at incubator for 3 hours, and then the medium was washed with 100 μl of DMSO. The absorbance was evaluated at 570 nm using a MiR plate reader. The experiments were repeated 3 times. 

### Apoptosis evaluation by using Annexin V-FITC apoptosis detection kit in dosimetry stage

Initially, 2×10^4^ SSCs and EL4 cellss were removed after treatment. Then, 500 μl of the binding buffer was added to the cell plate. In the next step, 5 μl of Annexin V-FITC and 5 μl of propidium iodide (PI) (21, 22) were added. At room temperature, it was incubated in foil for 10 minutes, then flow cytometry was performed and the percentage of apoptosis in both healthy and cancerous groups was evaluated.

### Evaluation of apoptotic gene expression

To determine the optimal dose of microRNA, after treatment of cancer cells, expression levels of apoptotic genes including *Bax, Bcl2 *and *Caspase 3* were evaluated by Q-PCR.

*Bax-*

F: 5ˊTGGGATGAATGGGGGAAGGGGAAA3ˊ

R: 5ˊAAGGGGACCTGAGGTTTATTGGCG3ˊ

*Bcl2-*

F: 5ˊATGGCGCAAGCCGGGAGAAC3ˊ

R: 5ˊCGCGTCCGCATCTCCAGCAT3ˊ

*Caspase 3-*

F: 5ˊCTCTGGTACGGATGTGGACG3ˊ

R: 5ˊCCCCTTCATCACCATGGCTT3ˊ

### Statistical analysis

To compare quantitative apoptosis data between different times, one-way analysis of variance (ANOVA) test was performed. To evaluate the effects of different cytotoxic concentrations of MiR at different times (group and time, and their interaction) as well as the effects of different concentrations of MiR at different times on the number of cells, two-way ANOVA was performed. Tukey’s multiple comparison was also performed. All data was analyzed by the software version 25. SPSS at level of P≤0.05.

## Results

### Cell culture and confirmation

**Fig.1:**
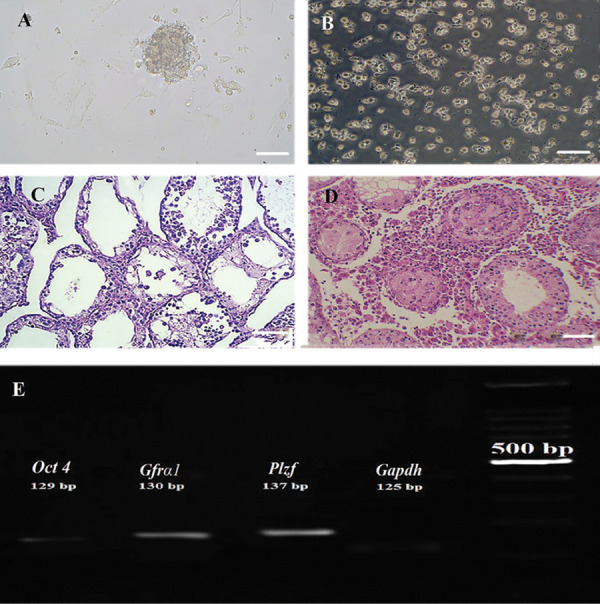
Cell culture and confirmation. **A.** The structure and nature of the cells of spermatogonia and cancer cells in the culture medium. Spermatogonia cells are colonized and sticky. **B.** Cancer cells have a rounded structure and a non-sticky nature. **C.** Tissue cross-sectional image of azoospermia mice, the structure of seminiferous tubules is observed without the presence of spermatogonia. **D. **Cross-section of mice testicular after tumor cell transplantation, the changing of the structure of the seminiferous tubules and infiltration of leukaemic cells are observed due to invasive tumor cells. **E. **Results of driving PCR products related to spermatogonia cells. The expression of (*Gapdh, Plzf, Gfrα-1, Oct4*) was proven by the reverse transcription polymerase chain reaction (RT-PCR) (scale bar: 100 µm).

SSCs were cultured in DMEM/F12 medium containing 2% fetal bovine serum (FBS, Gibco, USA) with glial cell line-derived neurotrophic factor (GDNF, Sigma, USA) 10 ng/ml for 2 weeks. Colony formation began after 24 hours (Fig .1A). EL4 cells were cultured in suspension (Fig .1B). The cell line was confirmed by flow cytometry analysis at the time of purchase as it was approximately 99% positive for EL4 cell-specific marker H2Kb (data not shown). Transplantation of EL4 cells confirmed their tumorigenicity potential as tissue histological examination revealed that the normal structure of the tubules had disappeared, and the leukemia cells had penetrated the interstitial tissue (Fig .1C, D). SSC was confirmed by expression of *Plzf, Gfrα-1* and *Oct4 *by PCR (Fig .1E). Additionally, based on flow cytometry analysis, the percentage of SSCs was 42.8% and 74.6% after one and two weeks of culture respectively (data not shown).

### Determination of particle size and charge with DLS

**Fig.2:**
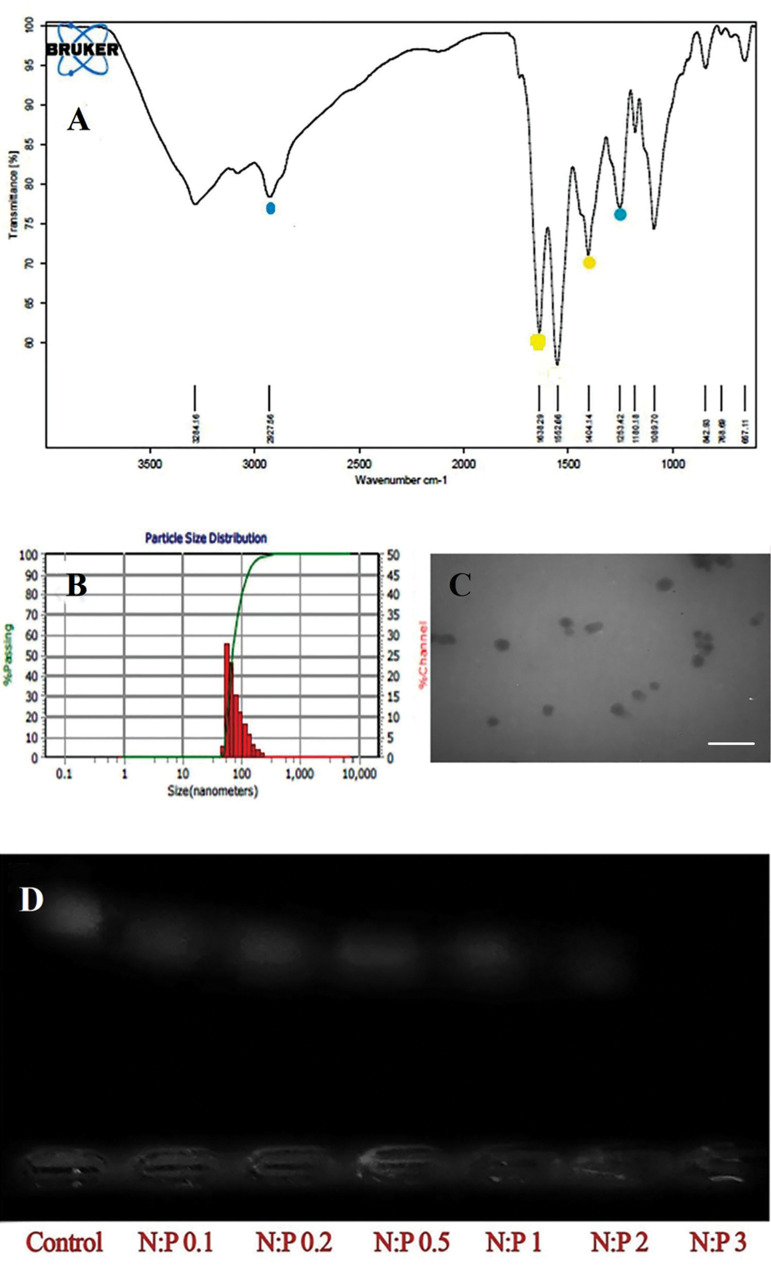
Nanoparticle evaluation tests. **A.** FTIR spectrum of polymer, folic acid and polymer coated with folic acid. The presence of the peaks of 1638 and 1404 (yellow spot) indexes of folic acid, 1263 and 2927 index loaded nanoparticles (blue spot). **B.** The particle size based on the DLS test. **C.** Transmission electron microscope showed a smooth and spherical surface in all nanoparticles. The average particle size was 69 nm (scale bar: 100 nm). **D.** Inhibition test for polyethylene amine electrophoresis gel.

To assure that the folic acid modification process on the surface of the prepared NPs was complete, the FTIR spectrum of polymer, folic acid, and polymer with folic acid are shown in Figure 2A. The specimens were measured in terms of particle size by using DLS. The surface load obtained in the Zeta bar in the sample of the loaded NP was -18 mv. The average particle size obtained with DLS was 69.8 nm (Fig .2B), and morphology by TEM showed a smooth and spherical surface in all NPs (Fig .2C). In order to meet a suitable mass ratio for 25 kDa polyethylene, we selected a 3:1 ratio (N:P equivalent of 20:1, Fig .2D).

### Toxicity of different doses MiR-PLGA-folic acid

SSCs and EL4 cells were incubated by different doses of NPs containing MiR 143 (25, 50 and 75 nmol) and MiR 206 (50, 100 and 150 nmol) at 24, 48 and 72 hours. There was no significant difference between the 24 hrs data and the control group and therefore this time was removed from our study. Additionally, data for 72 hours was less valuable than data for 48 hours due to cell doubling characteristics, so 48 hours was chosen as the optimal time. Each experiment was repeated 3 times.

**Fig.3:**
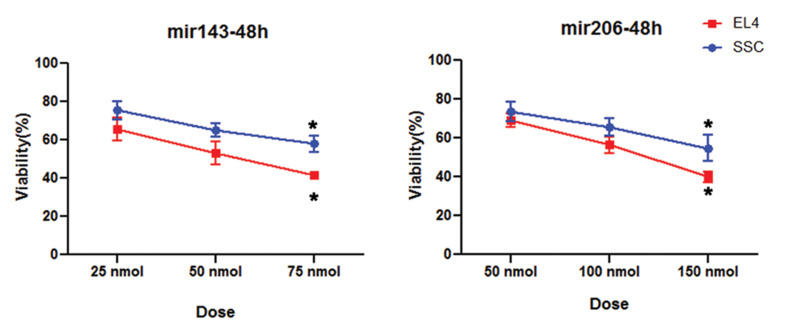
MTT test data. Based on MTT test, 48 hours after treatment with miR-PLGA-Acid folic, the highest rate of toxicity were observed in EL4 compared to SSC. *; Significant difference vs. other groups in the same cell (P≤0.05).

Based on MTT assay results, by two-way ANOVA, the optimal dose of MiR 143 was 75 nmol (59.87 ± 2.85% SSC and 35.3 ± 0.78 % EL4, P≤0.05) after 48 hours, and for MiR 206 it was 150 nmol (54.82 ± 6.7% SSC and 33.92 ± 3.01% EL4, P≤0.05) after 48 hours. In these doses, the survival rate of the EL4 cells and SSCs was below the half maximal inhibitory concentration (IC_50_) and above 50% respectively (P≤0.05, Fig .3).

### Determination of apoptosis rate at the optimal dose of microRNAs

**Fig.4:**
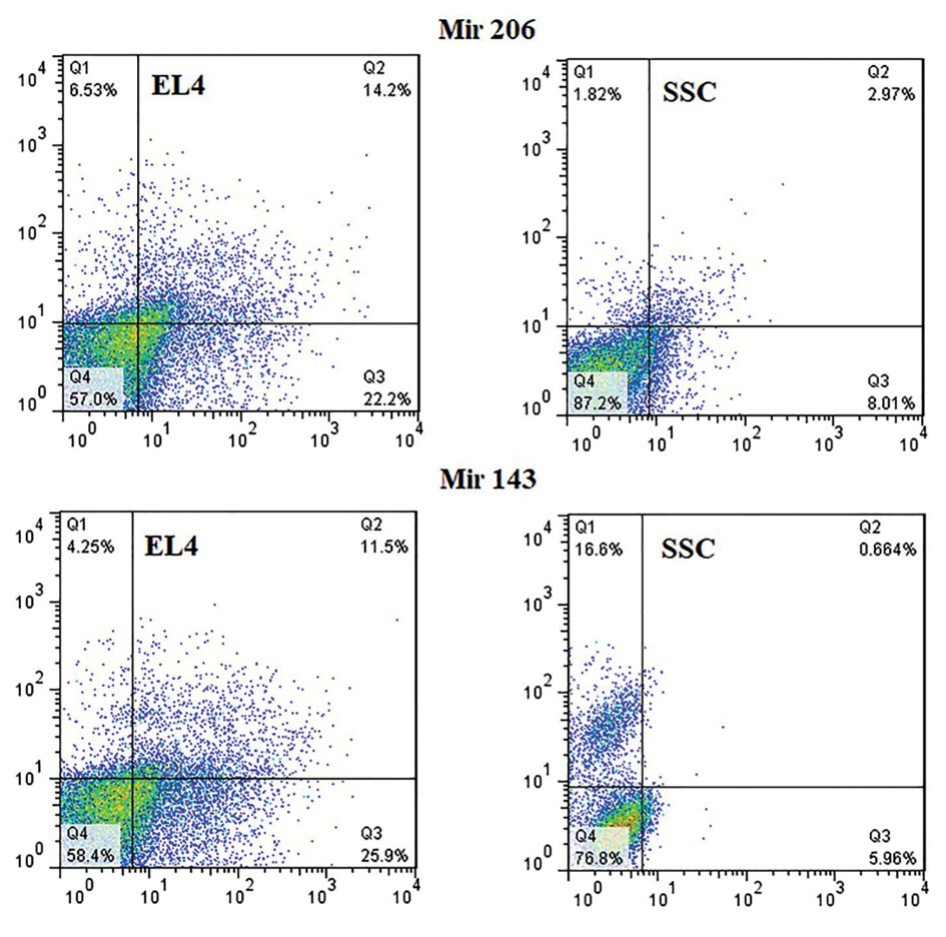
Based on the annexin assay, 48 hours after treatment with miR-PLGA-Acid folic, the highest rate of apoptosis were observed in EL4 compared to spermatogonial stem cell (SSC). The cells and microRNAs are shown separately in the image. Q1; Necrosis, Q2; Early apoptosis, Q3; Late apoptosis, and Q4; Survival rate.

**Fig.5:**
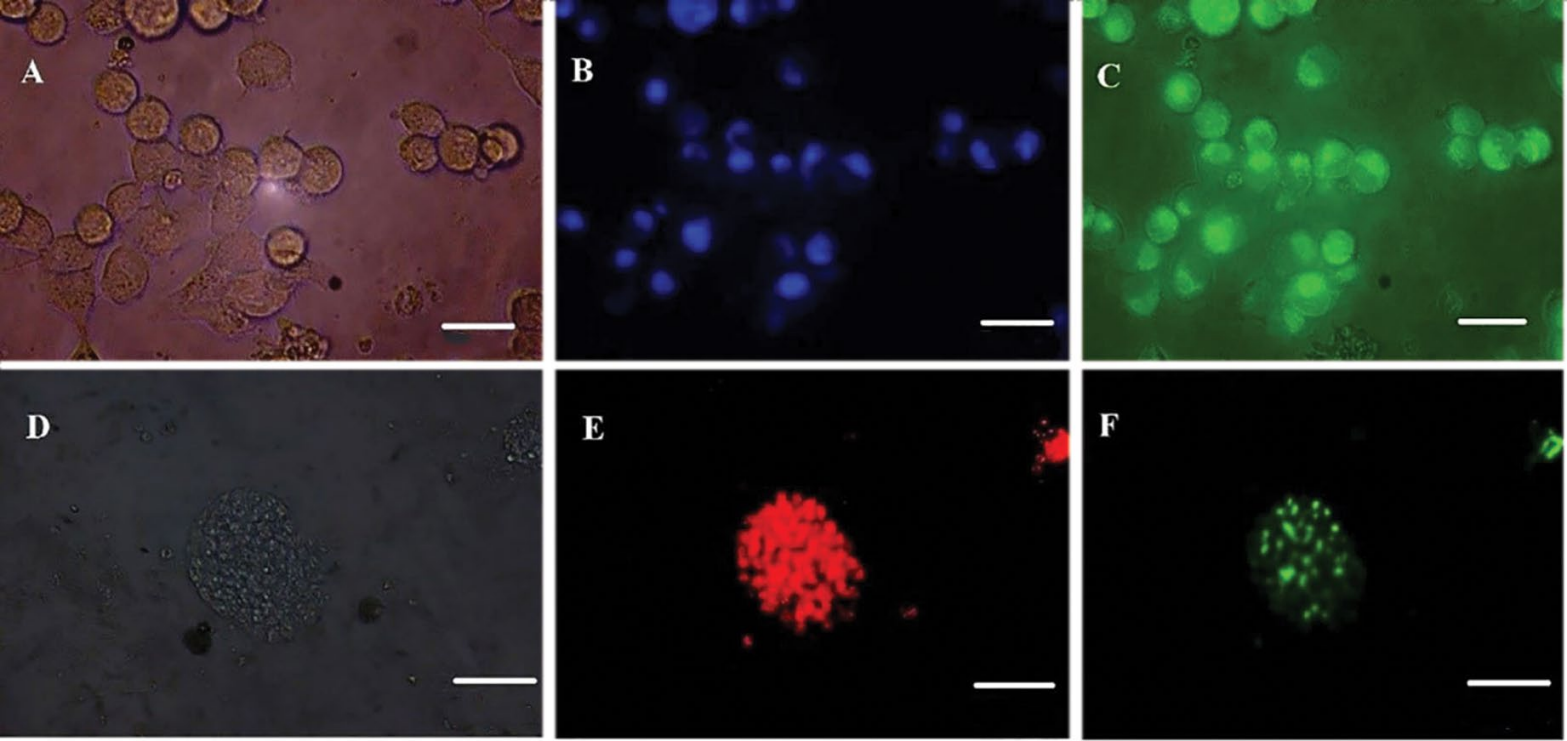
Fluorescent imaging of cells treated with PLGA-micro RNAs. **A.** Light microscope image of EL4 cells, **B. **Hoechst stained EL4 cells in blue, **C. **Fluorescent image of FAM-labeled green dyes inside EL4 cells at 570 nm wavelength, **D. **Spermatogonia colony, **E. **Plzf labeled spermatogonia, and **F. **Fluorescent image of FAM-labeled red dyes inside spermatogonial cells at 570 nm (scale bar: 50 μm).

Evaluation of apoptosis by the annexin kit also confirmed the optimal doses selected by MTT, for MiR 143, total apoptosis was (6.62 ± 1.8% for SSCs and 37.4 ± 1.2% for EL4 cells, P≤0.05), and for MiR 206, total apoptosis was (10.98 ± 1.5% for SSCs and 36.4 ± 3.7% for EL4 cells, P≤0.05) after 48 hours. In fact, using intelligent NPs at the same concentration, we were able to induce apoptosis in EL4 cells without any significant damage to the SSCs (Fig .4). Fluorescence microscopy images depict the extent of microRNA penetration into the cells (Fig .5).

Tukey’s multiple comparison test showed that *Bax* gene expression was significantly increased in cancer cells at the optimal dose of both microRNAs as compared to the control group (P≤0.04). *Bcl2* gene expression was decreased in both groups as compared to the control group, although there was no significant difference. There were significant correlations in the Mir 143 group (P≤0.01) and in the Mir 206 group (P≤0.048) as compared to the control group.* Caspase 3* expression was also significantly increased in the experimental groups (P≤0.01), but there was no significant relationship between the two treatment groups (Table 1).

**Table 1:** Expression of genes associated with the apoptosis process in EL4


PLGA-MiR treatment groups (48 hours incubation)	*Caspase 3*	*Bcl2*	*Bax*

PLGA-MicroRNA 143 treatment group	1.36 ± 0.2^a^	0.23 ± 0.02^a, b^	2.86 ± 0. 08^a, b^
PLGA-MicroRNA 206 treatment group	1.66 ± 0.15^a^	0.30 ± 0.01^a, b^	3.08 ± 0.03^ a, b^
Control	0.85 ± 0.04	0.49 ± 0.04	0.03 ± 0.01


Data are presented as mean ± SD. ^a^; There was a significant difference between the expression levels of the target gene in the treatment groups compared to control (P≤0.05), and,^ b^ ; There was a significant difference between two treatment groups in terms of gene expression (P≤0.05).

## Discussion

The importance of MiRs in cancer is highlighted by the observation that ∼50% of MiR genes are located in cancer-associated genomic regions or fragile sites (23). Studies have conclusively demonstrated that miRNAs are involved as tumor suppressors. In this study, we used two effective microRNAs, (143 and 206), to assess their effects on apoptosis in cancer cells. In our study, we showed apoptotic effects of these microRNAs on EL4 cells.

In this study, as in previous studies, we used the standard method of isolating and culturing spermatogonia cells and cancer cells, also, confirming them with appropriate PCR and flow cytometry methods (17, 18, 24).

In gene transfection, the method that should be used is the one that has the lowest toxicity, the highest level of performance and the lowest cost, while also having the ability to make a specific transfer (25). To overcome gene therapy problems and to improve efficiency, we used PLGA as the core and cationic PEI as a biodegradable shell.

We synthesized PLGA below 100 nm, which was a turning point in our study. In general, smaller NPs have a higher surface area to volume ratio than larger NPs, which increases the efficiency and interaction of NPs with the cell (26). In a study by Li et al. (27) with a size of 150 nm reached 70% absorbtion rate at 48 hours, In this study, with a nanoparticle size of approximately ≈ 70 nm based on flow cytometry data, we obtained a 62.54% uptake rate at 48 hours. Mohammadian et al. (28) also achieved the best result with a size of 15-60 nm at the same incubation time. The particles below 100 nm seem to be in best state for transfection (29). In agreement with other studies, the highest efficacy was observed after 48 hours of incubation (30, 31) that may increase at longer incubation times, only by non-selective passive uptake which is not desirable (32)

We also showed that with precise dosimetry, the NPs can prevent significant damage to SSCs. Folic acid, in the presence of a cell surface receptor, simply enters the cell and is absorbed through receptor-dependent endocytosis mechanism into the cell. Therefore, it is one of the most commonly targeted sites. Additionally, it is a preferred target because of many benefits, such as non-immunogenicity, small size, non-toxicity, and ease of handling (33). Due to the fact that, this receptor is expressed in cancer cells more than in healthy cells, by selecting folic acid as the targeting agent for nanoparticles, we tried to target cancer cells and preserve SSC, NPs moving towards cancer cells in order to preserve SSC, which we largely achieved. But at the same time, the small size of our NPs was like a double-edged sword, which was both more suitable for transfusion and increased peneteration into the cell. At the same time, it played a role in penetration of these NPs into SSC, which ultimately affected these cells. Certainly, further studies could help optimize this method to reduce non-selective influence.

We also benefited from the use of PEI as a watersoluble polymer for effective transfection. Because of high cationic charge at normal pH, this polymer is able to connect through a electrostatic bond to microRNA. Therefore, this is a valuable polymer for gene delivery, as demonstrated by other studies (34, 35).

In our study, various doses of MiR -PLGA 143/206 conjugated with folic acid were tested on tumor cells (EL4 cells) and healthy cells (SSCs). We chose doses based on previous studies, but we found a different effective dose. Our appropriate dose of each MiR was selected, based on the optimal decrease in EL4 cell survival and simultaneous SSC protection (i.e. less toxicity and apoptosis). The efficacy of therapeutic approach used in this study is similar to results reported in our perivous studies (17). In similar studies, the survival rate of cancer cells was reduced by increasing the time from 24 to 48 hours and increasing the dose (17, 28). Also, No studies have shown that treatment with a microRNA alone can completely eliminate cancer cells (36-38). In no similar study were both Mir 143 and 206 compared. In this study, based on MTT findings, we found a small difference in the survival rate of EL4 cells in the Mir 143 group which according to Mir 206 was more successful in inducing cell death, annexin assay did not show a significant difference. Both microRNAs appear to have the same effect in inducing cell death in cancer cells.

In this study, we showed that expression of pre-apoptotic gene *Bax2* and *Caspase 3* in cancer cell treared with PLGA-Mir was increased, and *Bcl2* expression decreased which is probably the mechanism of induction of apoptosis in cancer cells (39). A similar result was obtained in a previous study (17). As mentioned earlier, microRNAs activate apoptosis signaling in cancer cells by regulating apoptotic genes (39).

Due to the multivariate nature of cancer, (i.e. the existence of different microRNAs that play a role in either reducing or increasing cancer regulation), we need a method that can utilize the many potentials of microRNAs to control cancer. In this setting, miRNAs can be used as disruptors of cancer cells and sensitization agents, making the malignant mass more susceptible for the next line of treatment (40).

## Conclusion

In our first experience of utilizing MiRs for gene therapy on SSCs, we chose two MiRs because we were not certain about success rate,. MiR 206 has been demonstrated to have a tumor-suppressive role. According to this study and other similar studies, the efficacy of microRNAs in inducing apoptosis seems to be limited, and combinational therapy with medications may need to be considered for further efficacy. Taken together, this study suggests that MiR-therapy may lead to the development of novel therapeutic strategies for cancer, and apoptotic MiRs may be a potential therapeutic agent for human tumors and is worthy of further investigation. 
